# A Multi-Antigenic Adenoviral-Vectored Vaccine Improves BCG-Induced Protection of Goats against Pulmonary Tuberculosis Infection and Prevents Disease Progression

**DOI:** 10.1371/journal.pone.0081317

**Published:** 2013-11-21

**Authors:** Bernat Pérez de Val, Enric Vidal, Bernardo Villarreal-Ramos, Sarah C. Gilbert, Anna Andaluz, Xavier Moll, Maite Martín, Miquel Nofrarías, Helen McShane, H. Martin Vordermeier, Mariano Domingo

**Affiliations:** 1 Centre de Recerca en Sanitat Animal, Universitat autonoma de Barcelona–Investigación y tecnología Agroalimentarias, Campus de la Universitat Autònoma de Barcelona, Bellaterra, Catalonia, Spain; 2 TB Research Group, Animal Health and Veterinary Laboratories Agency-Weybridge, New Haw, Addlestone, Surrey, United Kingdom; 3 The Jenner Institute, University of Oxford, Old Road Campus Research Building, Roosevelt Drive, Oxford, United Kingdom; 4 Departament de Medicina i Cirugia Animals, Universitat Autònoma de Barcelona, Bellaterra, Catalonia, Spain; 5 Departament de Sanitat i Anatomia Animals, Universitat Autònoma de Barcelona, Bellaterra, Catalonia, Spain; University of Cape Town, South Africa

## Abstract

The “One world, one health” initiative emphasizes the need for new strategies to control human and animal tuberculosis (TB) based on their shared interface. A good example would be the development of novel universal vaccines against *Mycobacterium tuberculosis* complex (MTBC) infection. This study uses the goat model, a natural TB host, to assess the protective effectiveness of a new vaccine candidate in combination with Bacillus Calmette-Guerin (BCG) vaccine.

Thirty-three goat kids were divided in three groups: Group 1) vaccinated with BCG (week 0), Group 2) vaccinated with BCG and boosted 8 weeks later with a recombinant adenovirus expressing the MTBC antigens Ag85A, TB10.4, TB9.8 and Acr2 (AdTBF), and Group 3) unvaccinated controls. Later on, an endobronchial challenge with a low dose of *M. caprae* was performed (week 15). After necropsy (week 28), the pulmonary gross pathology was quantified using high resolution Computed Tomography. Small granulomatous pulmonary lesions (< 0.5 cm diameter) were also evaluated through a comprehensive qualitative histopathological analysis. *M. caprae* CFU were counted from pulmonary lymph nodes.

The AdTBF improved the effects of BCG reducing gross lesion volume and bacterial load, as well as increasing weight gain. The number of Ag85A-specific gamma interferon-producing memory T-cells was identified as a predictor of vaccine efficacy. Specific cellular and humoral responses were measured throughout the 13-week post-challenge period, and correlated with the severity of lesions.

Unvaccinated goats exhibited the typical pathological features of active TB in humans and domestic ruminants, while vaccinated goats showed only very small lesions. The data presented in this study indicate that multi-antigenic adenoviral vectored vaccines boosts protection conferred by vaccination with BCG.

## Introduction

Tuberculosis (TB), mainly caused *by Mycobacterium tuberculosis*, is one of the most important causes of infectious disease mortality and morbidity in humans worldwide [1]. Moreover, one-third of the world’s population is estimated to have latent TB infection (LTBI), making it one of the most prevalent human infections [2]. *M. bovis* and *M. caprae*, also members of the *M. tuberculosis* complex (MTBC), are the main causative agents of bovine and caprine TB, respectively. The latter is considered an emerging disease in a number of European countries, causing increasing economic losses to the livestock sector [3]–[5].

Goats infected with *M. caprae* may be a source of infection for cattle, acting as domestic reservoirs of bovine TB [6]. *M. caprae* has also been isolated from a wide range of wildlife species [4], [7], [8], and even from TB cases in humans [9]–[11]. However, in the European Union, there are currently no caprine TB control campaigns. In endemic areas, vaccination is seen as the best long-term prospect for TB control in livestock [12]. Reducing the disease prevalence prior to starting a test and sacrifice-based eradication program would reduce economic costs for the producers and the public sector.

Bacillus Calmette-Guerin (BCG), the only currently available vaccine, displays variable efficacy against human and animal TB [13]–[15]. In recent years new subunit vaccines have been developed to be used as boosters after a previous immunization with BCG or other live vaccines [16]. Viral delivery of such subunit vaccines has been widely used [17], [18]. Particularly, the use of adenoviruses as vectors for TB vaccines takes advantage on their natural tropism for the respiratory epithelium, as well as the strong immunity they induce [19], [20]. Boosting BCG with a recombinant replication-deficient adenovirus expressing the antigen Ag85A showed enhanced protection against TB in small laboratory animals [20], [21], cattle [22], [23], and goats [24].

Besides Ag85A, additional potential immunoprotective antigens are candidates to be included in multi-antigenic formulations. Among them, the MTBC antigens TB10.4 (Rv0288), TB9.8 (Rv0287) and Acr2 (Rv0251c) have recently been selected for this purpose on the basis of the induction of an early-CMI in calves after *M. bovis* infection of protected animals [25], and have been included in a new recombinant adenoviral vaccine named AdTBF. The effect of different doses and administration routes on the immune responses induced in cattle by BCG priming and AdTBF boosting have been recently assessed (G.S. Dean *et al*., unpublished data).

The aim of this study was to assess the effectiveness of a novel recombinant adenoviral booster vaccine expressing four MTBC antigens (Ag85A, TB10.4, TB9.8 and Acr2) to improve BCG protection against TB in goats challenged with *M. caprae*, their natural TB causing agent [26]. In addition to immunological evaluations and determination of bacterial load, exhaustive post-mortem analysis based on Computed Tomography (CT) and a comprehensive histopathological evaluation were used to assess vaccine efficacy.

## Materials and Methods

### 1. Study design

1. **Animals and experimental schedule.** Thirty-three female goat kids (3 month old Murciano-Granadina) obtained from an official TB-free herd of Murcia Region (South West of Spain) were selected on the basis of negative results to single intradermal comparative cervical tuberculin (SICCT) test and the Bovigam IFN-γ assay (Prionics, Schlieren, Switzerland). Subsequently, animals were randomly divided into three treatment groups of eleven goats each: Group 1, vaccinated with BCG; Group 2, vaccinated with BCG and boosted 8 weeks later with a recombinant adenovirus expressing a fusion protein containing four MTBC antigens (BCG-AdTBF); and Group 3, unvaccinated controls. Animals were observed daily for clinical signs and weighted every two weeks during the experiment.
2. **Ethics statement.** All animal experimental procedures were undertaken in accordance with the European Union Laws for protection of animals used in experimentation (86/609). Approval was obtained from the Animal Welfare Committee of the *Universitat Autònoma de Barcelona* and the *Generalitat de Catalunya* (Permit Number: 6332).
3. **Vaccines.** For the BCG inoculum preparation, *M. bovis* BCG Danish 1331 strain (ATCC, Ref. 35733™) was sub-cultured in Middlebrook 7H9 media (BD Diagnostics, Sparks MD, USA) supplemented with 0.5% (v/v) Tween 80, 40 mM sodium pyruvate (Sigma-Aldrich, Steinheim, Germany) and 10% (v/v) albumin dextrose catalase enrichment (BD Diagnostics). It was incubated for 28 days at 37°C. An aliquot of growth culture was titrated by platting 10-fold dilutions in phosphate buffered saline containing 0.05% Tween 80 (PBS-T80) on 7H11 media (BD Diagnostics) for 28 days at 37°C. The remaining aliquots were stored at –80°C prior to use. After bacterial count, growth culture was diluted to 10^6^ CFU/ml by suspension in phosphate buffered saline (PBS). A dose of 0.5 ml of this suspension was inoculated subcutaneously in animals of groups 1 and 2 at week 0 of the experiment.

The adenovirus type 5 construct AdTBF, which encodes Ag85A, TB10.4, TB9.8 and Acr2, was used at 1×10^9^ infectious units (iu) per animal and were injected intramuscularly in animals of group 2 eight weeks after vaccination with BCG.

1. **M. caprae challenge.** A field isolate of *M. caprae* SB0416 (www.Mbovis.org) was sub-cultured in Middlebrook 7H9 supplemented media at 37°C. After 28 days, an aliquot was platted on 7H11 media and cultured again for 28 days at 37°C and bacteria were counted as indicated above.

One week prior to challenge, goats were housed in Bio-Safety Level 3 boxes for acclimatization. Fifteen weeks after BCG vaccination, all animals were anesthetized with 4–6 mg/kg of propophol (Propofol Lipuro®) and 0.2 mg/kg of midazolam (Dormicum®) administrated intravenously. Subsequently the animals were challenged through the endobronchial route with a suspension of approximately 1.5×10^3^ CFU of *M. caprae* as previously described [27].

### 2. Post-mortem studies

All goats were euthanized by administration of an intravenous sodium pentobarbital overdose at week 13 after *M. caprae* challenge, i.e. week 28 of the experiment. At necropsy, pulmonary lymph nodes (LN) were aseptically removed making sure the pleural lung surface was not sectioned, and the volume of gross lesions and bacterial load were measured. The number, extension, and distribution of TB lesions in the lungs were recorded visually and quantified by Computed Tomography (CT). Microscopic features of small lung granulomatous lesions were assessed by histopathological examination.

**2.1. Lungs.** Lungs were fixed *in toto* by perfusion with 10%-buffered formalin through the trachea while being held vertically. After complete lung distension by the fixative, the trachea was tied, and whole lungs were immersed into formalin filled containers. Six month later, fixed lungs were scanned with a high resolution 64-slice Multi-Detector CT scan (Brillance CT 64-channel, Philips Medical Systems, Cleveland, Ohio, USA), and sequential slices were analyzed on a work station (Aquarius Station, TaraRecon, Foster City, California, USA) as previously described [27]. This procedure, allowed the calculation of the total volume of granulomatous lesions relative to the whole lung volume.

Once scanned by CT, lungs were sliced in approximately 1 cm thick sections to visually examine the lesions. Animals were classified in three groups depending on the maximum diameter of the granulomas found in their lungs. Three granuloma diameter size intervals were defined: < 0.5 cm, Small Granulomas (SG); 0.5–2 cm, Medium Granulomas (MG); and 2 cm, Large Granulomas (LG). The number of affected lung lobes was also recorded.

**Figure 1:**
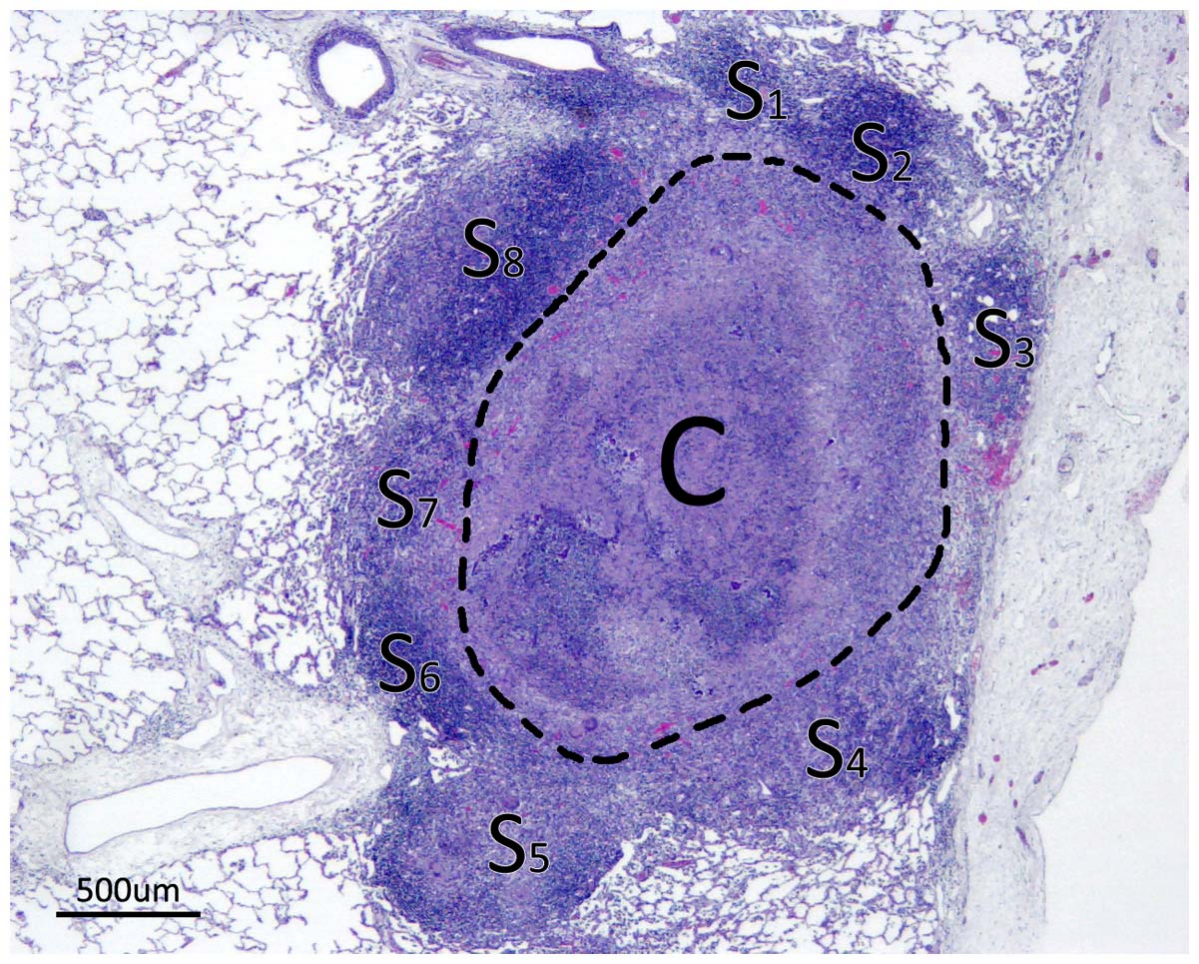
Scheme of a granuloma surrounded by satellite granulomas (SatG). Example of TB small lesion in lung consisting of a central granuloma (C) surrounded by 8 smaller SatG (S_1−8_) characterized by variably sized clusters of inflammatory cells contiguous to the central granuloma, sometimes with presence of MNGC and central necrosis (e.g. S_5_). The number of SatG, upon histopathological assessment of small granulomas, was used as a parameter to evaluate the tendency of lesions to disseminate.

Representative LG and MG from each goat (when present) were investigated histopathologically. In addition, five SG from each animal (with the exception of a goat of the BCG-AdTBF group which presented only 4 SG) were also selected for histopathological study to determine possible qualitative morphological differences between groups. Granulomas were sectioned through their maximum diameter and embedded in paraffin. Then, 4 µm sections were stained with hematoxylin and eosin (HE). Slides were microscopically examined in parallel by two pathologists (blinded regarding the experimental groups). The following parameters were scored: 1) Stage of granuloma development according to the four stages previously defined by Wangoo *et al*.: Stage I (Initial), Stage II (Solid), Stage III (Minimal necrosis) and Stage IV (Necrosis and mineralization) [28]; 2) number of satellite granulomas (SatG) surrounding the central lesion (a descriptive scheme is shown in Figure 1); 3) presence of multinucleated giant cells (MNGC) as previously described [29]: 0, 1 to 10 and > 10 MNGC; 4) central necrosis extension (absence of necrosis, necrosis comprising < 50%, and ≥ 50% of granuloma surface); and 5) mineralization degree: absence, low and high.

**2.2. Pulmonary LN.** Gross lesions in pulmonary LN (caudal and cranial mediastinal, right and left tracheobronchial) were measured as previously described [27]. The volume of visible lesions of each animal was calculated as 4/3×π×*r* ^3^ (where *r* is the lesion radius) taking into account the sphere-like morphology of the lesions found. Recording of all granuloma diameters was performed by the same pathologist in order to ensure the same criterion was followed for all samples.

After gross pathological evaluation, whole pulmonary LN were homogenized and decontaminated as previously described [27]. The total viable bacterial count in LN was determined by plating 0.1 ml of serial dilutions of LN homogenates on 7H11 agar plates (BD Diagnostics). The inoculated media were incubated at 37°C for 28 days. After that, the total CFU count of each LN was calculated.

### 3. Assessment of immune responses

1. **Antigens and peptides.** Bovine tuberculin (PPD-B) and avian tuberculin (PPD-A) were obtained from CZ Veterinaria (Porriño, Spain). Recombinant mycobacterial proteins TB9.8, TB10.4, Ag85A, Acr2 and MPB83 were obtained from Lionex (Braunschweig, Germany). Peptide cocktails ESAT-6/CFP-10 (E/C) and Rv3615c [27], were supplied by AHVLA.
2. **IFN-γ Whole blood assay.** Blood samples were collected from the jugular vein in heparinized blood tubes. One ml of whole blood was stimulated in 96-deepwell cell culture plates (Eppendorf Ibérica, Madrid, Spain) with PBS, PPD-B at 10 µg/ml, Ag85A, E/C and Rv3615c at 5 µg/ml, or phytohemagglutinin (Sigma-Aldrich) at 10 µg/ml. Plasma supernatants were collected after 24h of culture at 37°C and 5% CO_2_ and stored at –20°C until tested by Bovigam IFN-γ enzyme-linked immunosorbent assay (ELISA), which was performed according to the manufacturer’s instructions. ELISA results were obtained as Optical Density determined at 450 nm (OD_450_). Specific reaction was expressed as ΔOD_450_ (OD_450_ of antigen-stimulated sample minus OD_450_ of non-stimulated control). The assay was performed every two weeks throughout the experiment using PPD-B and Ag85A as stimuli. Peptide cocktail E/C and Rv3615c were also included at weeks 15, 20, 26 and 28.
3. **IFN-γ Cultured ELISPOT assay.** At week 15 (prior to challenge), Peripheral Blood Mononuclear Cells (PBMC) were isolated from blood of a subset of 10 randomly selected vaccinated goats (5 of BCG group and 5 of BCG-AdTBF group). Blood samples were diluted 1:1 in PBS, separation of blood cell populations was performed with a gradient centrifugation using Histopaque-1077 (Sigma-Aldrich). PBMC were stimulated in 24-well plates (2×10^6^ cells/well) with 10 µg/ml of PPD-B in cell culture medium: RPMI 1640 medium (Sigma-Aldrich) supplemented with 10% fetal calf serum (Sigma-Aldrich), non-essential amino-acids (Sigma-Aldrich), 5×10^−5^ M 2-mercaptoethanol, 100 U/ml penicillin and 100 μg/ml streptomycin sulphate. Cells were incubated at 37°C 5% CO_2_. Cultured ELISPOT assay was based on a previously described method [23], briefly, recombinant human IL-2 (Sigma-Aldrich) was added to cell cultures at a final concentration of 10 U/ml at days 5 and 8. Half of supernatant was replaced with fresh cell culture medium without IL-2 at days 10 and 12. On day 13, 5×10^4^ cultured cells/well were added into ELISPOT plates (MultiScreen HTS, Merck Millipore, Darmstadt, Germany) previously coated overnight at 4°C with bovine monoclonal IFN-γ antibody (Acris Antibodies GmbH, Herford, Germany), which recognizes goat IFN-γ, and then blocked with 10% fetal calf serum in RPMI medium. Cells were stimulated with 5 μg/ml of Ag85A, TB10.4 and TB9.8 in separated wells, and incubated 24h at 37°C 5% CO_2_. Cultures were performed in the presence of autologous antigen presenting cells (obtained from PBMC isolated at week 15, as described in [30]). After that, biotin-labelled bovine monoclonal IFN-γ antibody (Acris Antibodies GmbH), which also recognizes goat IFN-γ, and phosphatase-conjugated streptavidin (Life Technologies S.A., Madrid, Spain) were added to develope the spots. Spot-Forming Cells (SFC) were revealed and counted as previously described [23], and the number of SFC/ml was calculated.
4. **Serological tests.** Humoral responses to vaccination and *M. caprae* infection were studied by carrying out IgG indirect ELISAs to the MTBC protein MPB83 and the four antigens expressed by the adenoviral vaccine (Ag85A, Acr2, TB9.8 and TB10.4). Ninety-six-well plates were coated for each of the five antigens separately (Nunc Maxisorp; Thermo Fisher Scientific, Roskilde, Denmark) at a final concentration of 0.5 µg/ml each, diluted in carbonate/bicarbonate buffer. The plates were incubated overnight at 4°C. For the MPB83-specific ELISA, plasma samples from all goats were analysed at weeks 0, 4, 8, 15 and then every two weeks, whereas for the four AdTBF-antigens ELISAs plasma samples were analyzed at weeks 0, 8, 9, 10 and then every two weeks. The IgG ELISAs were performed as described previously [24]. PPD-B and PPD-A intradermal inoculations were performed at week 26 following the standard procedures for SICCT test in order to generate a boost-effect on humoral responses at week 28.

### 4. Statistical analyses

One-way ANOVA with Student-Newman-Keuls multiple comparison test was used for comparisons among groups in terms of differences in weight increase, volume of lesions, number of affected lobes, number of SatG and bacterial load (log_10_-transformed data). Whole blood IFN-γ responses and serological tests were compared by non-parametric Kruskal-Wallis test with *post hoc* Mann-Whitney or Wilcoxon test, whereas IFN-γ SFC differences between vaccinated groups were compared by Student’s unpaired two-sample T-test. Differences in observed frequencies of qualitative histopathological features among groups were assessed by chi-squared test. Correlations between post-mortem parameters, as well as between IFN-γ cultured ELISPOT results and post-mortem parameters were assessed by using Pearson’s correlation, whereas the rest of immunological responses were compared with post-mortem parameters by employing non-parametric Spearman rank test. Data analysis was performed using SPSS statistical package version 19.0 (IBM Inc. Chicago, IL, USA).

## Results

### 1. Clinical signs and body weight

**Figure 2:**
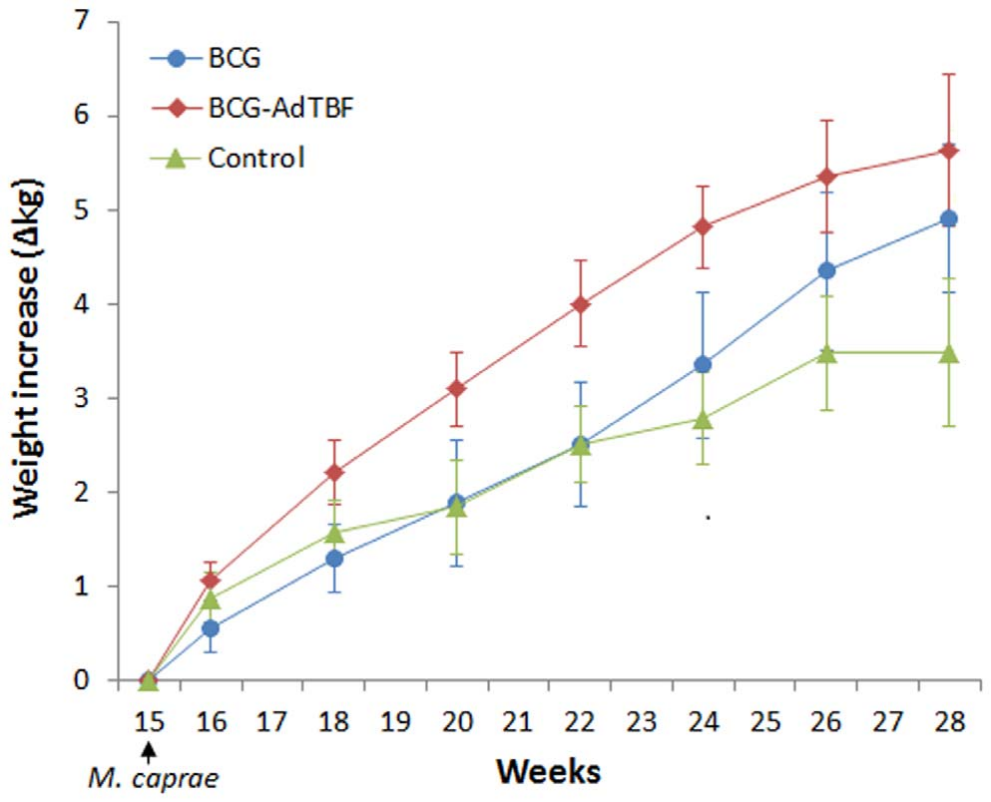
Body weight after *M. caprae* infection. Goats were challenged with 1500 CFU *M. caprae* at week 15 and weighted weekly untill the end of the experiment (week 28). Each line represents the cumulative weight increases (in kg ± 95% CI) in the three treatment groups from the challenge to the end point.

Clinical signs typical of caprine TB were monitored from the *M. caprae* challenge to the end point of the study. Coughing was observed in 7/11 BCG, 2/11 BCG-AdTBF and 6/11 unvaccinated control goats. The mean body weight increase during this period was significantly lower in unvaccinated control animals (249 g/week, 95% CI: 230-269) in comparison to BCG-AdTBF group (403 g/week, 95% CI: 383-423, *P<*0.001) and BCG group (351 g/week, 95% CI: 331-370, *P<*0.05). The weight increase during the post-challenge period of the different groups is shown in Figure 2.

### 2. Gross pathology and bacteriology

All animals presented macroscopic TB lesions at necropsy exhibiting a wide range of sizes between groups. Large lesions often developed conspicuous liquefaction and cavities. TB lesions were mainly restricted to the respiratory system, with the exception of 4 unvaccinated goats which also showed extra-pulmonary TB lesions in liver (n = 3), pericardium (n = 2) and spleen (n = 1).

**Figure 3:**
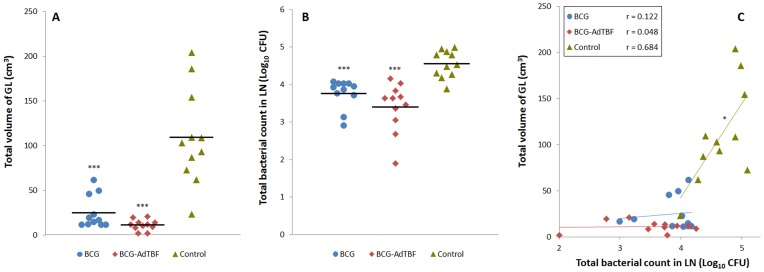
Quantification of pulmonary pathology and bacterial burden. (A and B) The sum of gross lesions (GL) volumes in lungs (measured by CT) and thoracic LN (measured by direct visual examination) is expressed in cm^3^, and the total bacterial counts in thoracic LN are expressed as Log_10_-transformed CFU. Horizontal lines indicate mean values. ***P<0.001, one-way ANOVA/Student-Newman-Keuls multiple comparison test. (C) Correlation between the volume of GL and bacterial counts. Results are divided according to treatment groups (BCG, circles; BCG-AdTBF, diamonds; and Control, triangles). *P<0.05, Pearson’s correlation (r).

The sum of gross lung and LN lesion volume and the bacterial load in respiratory LN, are shown in Figure 3 (A-B). Vaccinated groups showed a significant reduction of gross lesions (mean volume of gross lesions) and bacterial load (mean Log_10_ CFU/LN) in comparison to the unvaccinated group (*P<*0.001). Significant differences were also found in the volume of gross lesions between the two vaccinated groups. The BCG-AdTBF group showed lower volume of gross lesions in lungs (mean Log_10_ cm^3^: 0.9, 95% CI: 0.7-1.2; *P<*0.05) and LN (mean Log_10_ mm^3^: 2.3, 95% CI: 2.1-2.5; *P<*0.05) compared with BCG group (mean Log_10_ cm^3^: 1.3, 95% CI: 1.1-1.4; and mean Log_10_ mm^3^: 2.9, 95% CI: 2.6-3.2; respectively). A direct correlation between bacterial load and total volume of gross lesions was only found in unvaccinated animals (r  =  0.684, *P<*0.05, Figure 3C). In vaccinated animals, however, the gross lesion volume reduction was more pronounced than was the bacterial load reduction.

The extension of gross lesions in the lung was also assessed to classify animals depending on the size of the biggest granuloma found in their lungs (maximum granuloma size). Ten out of 11 unvaccinated goats developed one or more LG, whereas these granulomas were found only in 1 BCG and 1 BCG-AdTBF vaccinated goats. Eleven animals showed at least one MG and no LG (6 BCG, 4 BCG-AdTBF and 1 control) and, finally, ten vaccinated goats (4 BCG and 6 BCG-AdTBF) showed only SG. These differences in maximum granuloma size between vaccinated and unvaccinated groups were statistically significant (*P<*0.001).

**Figure 4:**
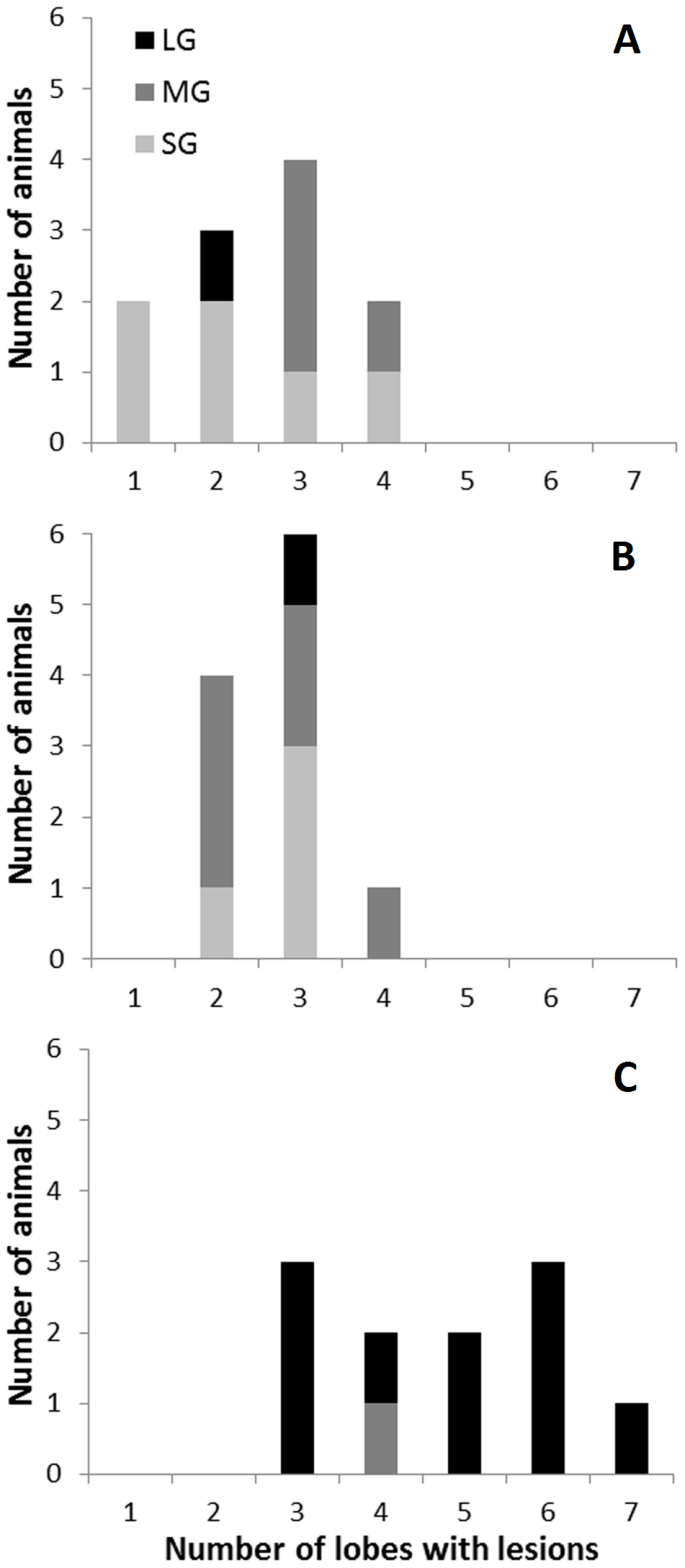
Extension of gross lesions and distribution in the lungs. Individual relationship between the number of lung lobes with TB lesions (out of 7) and the classification according to the maximum sized granuloma found in the lungs (SG, Small Granuloma: < 0.5 cm diameter, clear grey; MG, Medium Granuloma: 0.5–2 cm diameter, grey; and LG, Large Granuloma: > 2 cm diameter, black). Results are divided according to the three groups: BCG-AdTBF (A), BCG (B) and Control (C).

Vaccinated goats also presented lower lesion dissemination amongst lung lobes. The mean number of affected lung lobes was of 2.7 (95% CI: 2.3-3.2) in the BCG group and of 2.5 (95% CI: 1.9-3.2) in the BCG-AdTBF group, both of them significantly lower than that found in the control group (4.7, 95% CI: 3.8-5.7, *P<*0.001). The distribution of goats in each group with regard to maximum granuloma size classification and the dissemination of gross lung lesions (number of affected lobes) are shown in Figure 4.

### 3. Histopathological analysis

**Figure 5:**
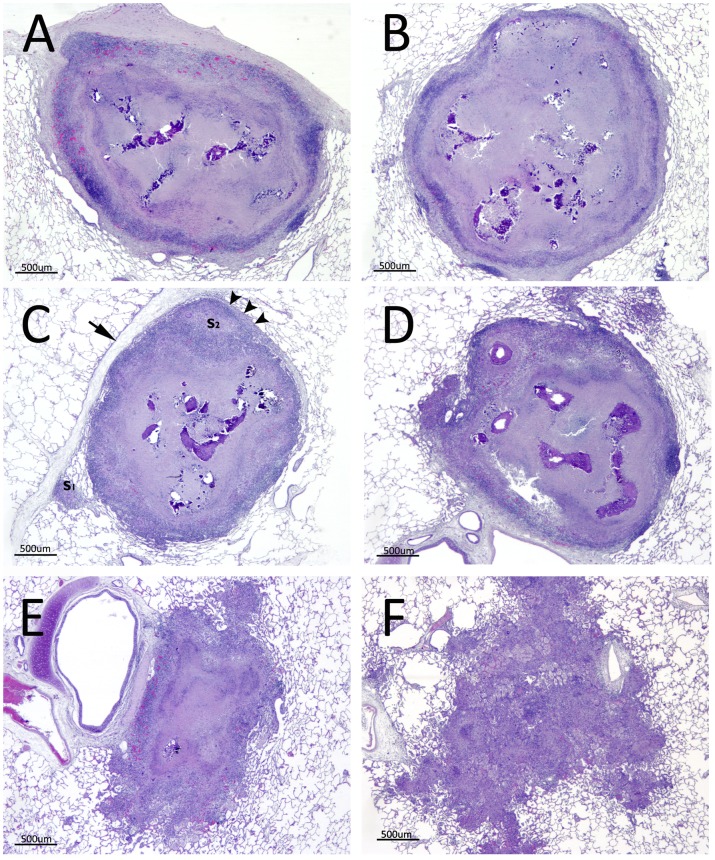
Staging of small granulomas (SG) by histopathological analysis. Representative developmental stages of the SG found in formalin-fixed lungs. (A-B) Stage IV. SG from 2 BCG-vaccinated goats. Encapsulation is complete. Extensive central necrosis with high degree of mineralization. (C-D) Stage III. SG from 2 BCG-AdTBF-vaccinated goats. Incomplete encapsulation. Macrophages and lymphocytes surrounding the central necrosis and mineralization. Note the formation in C of 2 satellite granulomas (SatG, S_1_ and S_2_). S_2_ is surrounded by a thin mantle of fibroblasts (arrowheads) which is connected to the intralobular septa (arrow). (E) Stage II. SG from an unvaccinated goat, presenting an irregular outline, central necrosis without mineralization and SatG formation. (F) Stage I. SG from an unvaccinated goat. Unstructured lesion presenting a diffuse mixture of inflammatory cells, without a distinguishable necrotic core.

There were no evident structural differences among groups with regard to LG and MG. Typical advanced granulomas were seen, showing prominent and multifocal caseous necrosis, extensive areas of mineralization, and irregular margins with epithelioid cells and some MNGC surrounding the necrosis. In contrast, qualitative structural differences were found in SG among groups (Table 1). A total of 164 SG were analyzed (55 from BCG group, 54 from BCG-AdTBF group and 55 from Control group). Vaccinated goats had a higher proportion of SG with higher grade of necrosis (83%, *P<*0.05), as well as more mineralization (38%, albeit not statistically significant) in comparison to unvaccinated controls (64% and 20% respectively). BCG and BCG-AdTBF groups showed a significantly higher frequency of SG in advanced developmental stages (IV and III respectively, *P<*0.05). Examples of representative SG of vaccinated groups are shown in Figure 5 (A-D). By contrast, unvaccinated animals presented a higher proportion of SG at early stages (24% at stages I and II), showing poorly delimitated microscopic lesions with a higher number of SatG (51% with ≥ 4 SatG, *P<*0.01, Figure 5E-F). The mean number of SatG in each animal correlated positively with both the volume of gross lesions and the number of affected lung lobes (r  =  0.418, *P<*0.05; r  =  0.546, *P<*0.01; respectively).

**Table 1:** Histopathological analysis of pulmonary small granulomas (< 0.5 cm diameter).

		No. of granulomas per group (%)		
Granuloma feature	Value	BCG^a^	BCG-AdTBF^b^	Control^a^
**Necrosis**				
	Low or none	10 (18)	9 (17)	20 (36)
	High	45 (82)*	45 (83)*	35 (64)
**Mineralization**				
	Low or none	34 (62)	34 (63)	44 (80)
	High	21 (38)	20 (37)	11 (20)
**Developmental stage**				
	I	1 (2)	0 (0)	1 (2)
	II	6 (11)	6 (11)	12 (22)
	III	25 (45)	38 (70)*	26 (47)
	IV	23 (42)*	10 (19)	16 (29)
**No. of MNGC** c				
	< 10 or none	23 (42)	10 (19)	16 (29)
	≥ 10	32 (58)	44 (81)*	39 (71)
**No. of satellite granulomas**				
	None	15 (27)	9 (17)	7 (13)
	1–3	28 (51)	28 (52)	20 (36)
	≥ 4	12 (22)	17 (31)	28 (51)**

a Five granulomas /animal (N = 55).

b One animal only presented 4 granulomas (N = 54).

c MNGC: multinucleated giant cells.

* *P<*0.05, ** *P<*0.01 (Chi-squared test).

**Figure 6:**
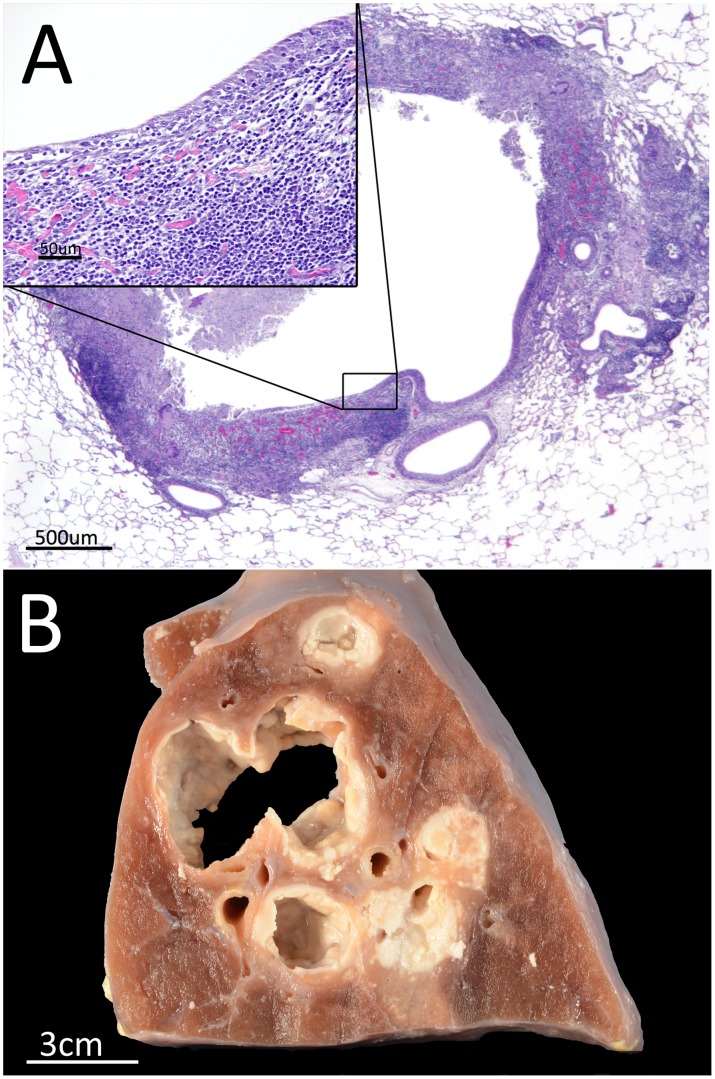
Progression of caseous necrosis to cavitary lesions. (A) The caseous necrosis in the centre of the granuloma progresses to liquefaction and, if not effectively encapsulated, the necrotic reaction destroys the epithelium of an adjacent bronchiole, emptying the content of the liquefied lesion into the airways. (B). Large cavitation of pulmonary parenchyma, originates from progressive liquefaction of confluent granulomatous lesions, observed macroscopically in all unvaccinated goats.

Vaccinated animals showed a higher proportion of SG at later stages with partial or complete encapsulation (i.e. stages III and IV, Figure 5A-D). These lesions showed highly organized structure containing a very low proportion of lymphocytes and macrophages surrounding the central necrotic area. On the other hand, microscopic lesions observed in many unvaccinated animals showed SG at early-stages, with a poorly organized mixed inflammatory infiltrate composed of lymphocytes, macrophages and neutrophils and lower necrosis extension (see Figure 5E-F). Unvaccinated goats frequently showed SG which opened to the bronchiolar lumen, emptying its content into the airways leading to the formation of small cavities (Figure 6).

### 4. Immune responses following vaccination and challenge

**Figure 7:**
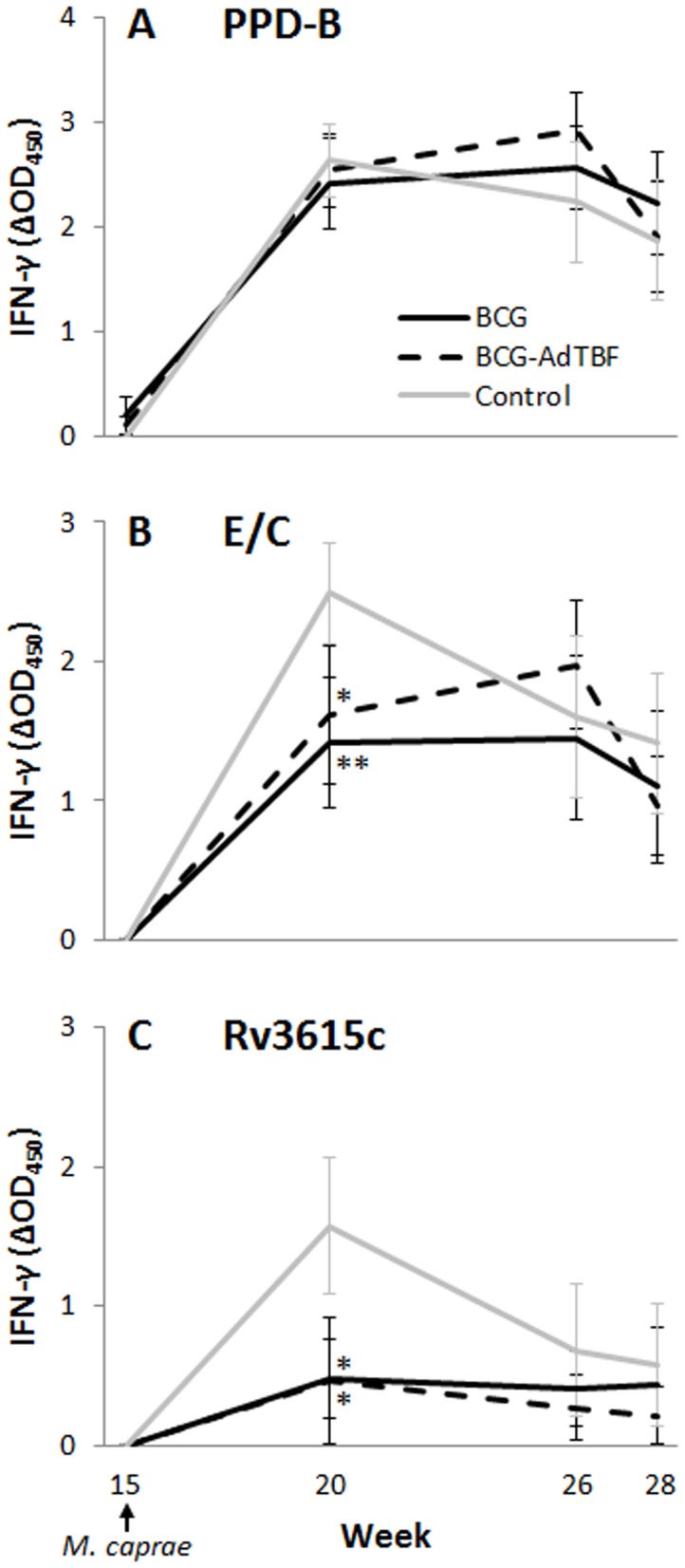
Antigen-specific IFN-γ responses after *M. caprae* challenge. Determination by ELISA of the IFN-γ released after peripheral blood stimulation with: (A), bovine PPD (PPD-B); (B), ESAT-6/CFP-10 peptide cocktail (E/C); and (C), Rv3615c protein. The results are expressed as mean ΔOD_450_ ± 95% CI for the 3 treatment groups (BCG, BCG-AdTBF and Control). *P<0.05, **P<0.01, Kruskal-Wallis/Mann-Withney post-hoc test.

The whole blood IFN-γ responses to E/C and Rv3615c-specific showed significant differences among groups at week 20, moment at which the peak response was reached in unvaccinated animals (Figure 7). At this time point, the mean E/C-specific IFN-γ response in the control group was significantly higher than in the BCG (*P<*0.01) and BCG-AdTBF (*P<*0.05) groups. Also, the mean Rv3615c-specific IFN-γ response in the control group was significantly higher than in both vaccinated groups (*P<*0.05). At the rest of time points, responses were not significantly different among groups.

IFN-γ responses to PPD-B and Ag85A were followed-up throughout all the experiment. Moderate PPD-B-specific IFN-γ responses were detected from 2 weeks after BCG vaccination up to the challenge point (week 15) in the two vaccinated groups (data not shown). A dramatic increase of IFN-γ responses to PPD-B was observed in the three experimental groups after *M. caprae* challenge. These responses were maintained up to the end of the experiment without significant differences among groups (Figure 7). Ag85A-specific IFN-γ responses were undetectable throughout the experiment until two weeks after AdTBF inoculation (week 10), when they were significantly higher in the BCG-AdTBF group (mean ΔOD: 0.085, 95% CI: 0.021-0.149) compared with BCG (mean ΔOD: 0.025, 95% CI: 0-0.05, *P<*0.05) and control (mean ΔOD: 0.007, 95% CI: 0.04-0.01, *P<*0.01) groups.

*Ex vivo* IFN-γ responses to E/C and Rv365c were assessed as correlates of disease progression. At week 20 of the experiment (when significant differences among groups were found), IFN-γ responses to both E/C and Rv3615c correlated positively both with the total volume of gross lesions (Spearman’s ρ: 0.388, *P<*0.05; 0.519, *P<*0.01; respectively) and with the bacterial load (Spearman’s ρ: 0.559, *P<*0.001; 0.4, *P<*0.05; respectively).

**Figure 8:**
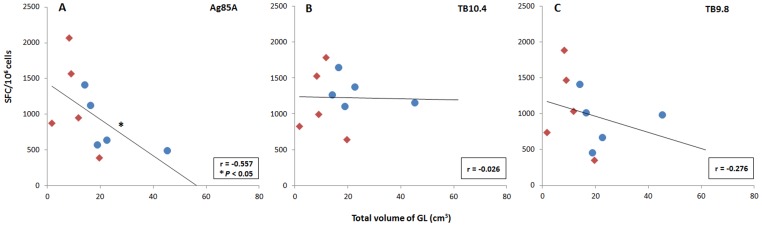
Vaccine-induced memory T cells correlated with infection outcome. IFN-γ cultured ELISPOT responses to 3 AdTBF antigens (Ag85A, TB9.8 and TB10.4) performed prior to *M. caprae*-challenge (week 15) in 10 vaccinated goats (5 BCG [•] and 5 BCG-AdTBF [⧫]). Figure shows the correlations of Spot Forming Cells (SFC) with the total volume of gross lesions (GL) measured post-mortem. *P<0.05, Pearson’s correlation.

Results of the IFN-γ cultured ELISPOT performed prior to challenge (week 15) were also compared with post-mortem parameters. Figure 8 shows the correlations between IFN-γ antigen-specific SFC and the total volume of gross lesions in 10 vaccinated goats (5 from BCG group and 5 from BCG-AdTBF group). Only Ag85A-specific SFC showed significant inverse correlation with gross lesions (r  =  –0.557, *P<*0.05). Even though without statistical significance, slightly inverse correlation of SFC specific to TB9.8 with the volume of gross lesions was also obtained (r  =  –0.276). No evident correlation was found between cultured ELISPOT results and bacterial counts (data not shown).

**Figure 9:**
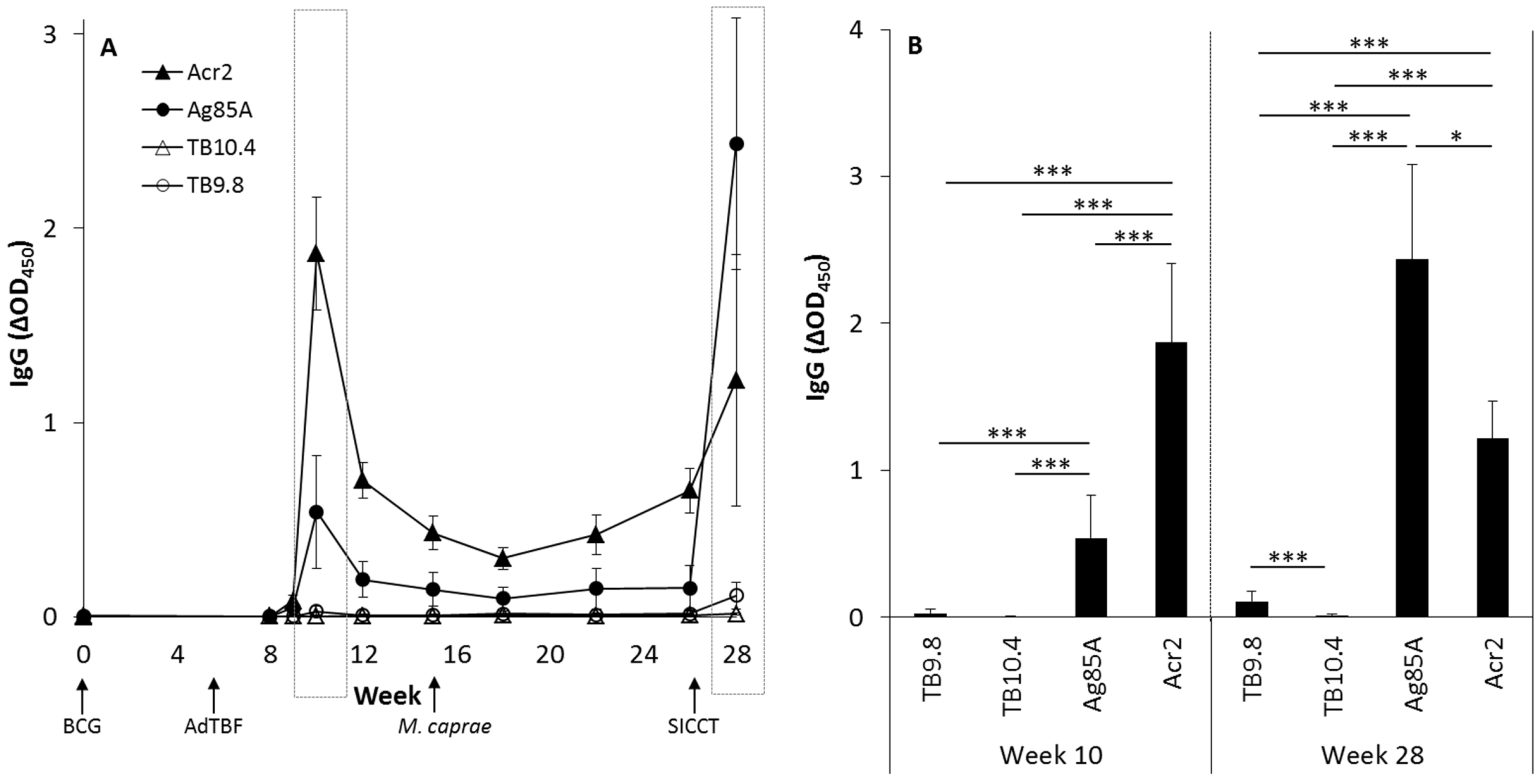
Vaccine and challenge-induced antibody responses. (A) Kinetics of the IgG specific responses in the 11 goats primed with 5×10^5^ CFU BCG (week 0), boosted with 10^9^ PFU AdTBF (week 8) and challenged with 1500 CFU *M. caprae* (week 15), to the four AdTBF antigens (Acr2, Ag85A, TB10.4 and TB9.8). SICCT: single intradermal comparative cervical tuberculin test (week 26). The dashed boxes indicate the IgG responses at weeks 10 and 28. (B) Comparison of IgG responses at 2 weeks after AdTBF immunization (week 10), and 2 weeks after SICCT test (week 28). *P<0.05, ***P<0.001, Kruskal-Wallis/Wilcoxon post-hoc test.

Mean IgG responses to AdTBF-encoded antigens in BCG-AdTBF prime-boosted goats are shown in Figure 9. Two peaks were observed, one at two weeks after AdTBF-immunization and the second two weeks after PPD-B and PPD-A intradermal inoculations (weeks 10 and 28 respectively, Figure 9A). At these time points significant differences were found among the different antigen-specific antibody responses (Figure 9B). Humoral responses to AdTBF-encoded antigens were undetectable in the other two groups throughout the experiment.

The antibody responses to *M. caprae* were measured as MPB83-specific IgG. At week 20, a peak of IgG levels was found in the control group (mean ΔOD 1.2, 95% CI: 0.2-2.1), whereas IgG levels in vaccinated animals were practically undetectable in the two vaccinated groups (both with mean ΔOD: 0.01, 95% CI: 0-0.03) and, thus, were significantly lower compared with the control group (*P<*0.001). At this time point, the IgG responses to MPB83 correlated positively with the total volume of gross lesions (Spearman’s ρ: 0.524, *P<*0.01) and with the bacterial load (Spearman’s ρ: 0.481, *P<*0.01). All animals presented higher IgG levels at week 28 (after the intradermal tuberculin boost effect) and statistically significant differences were not found among the three groups (Control group mean ΔOD: 2.9, 95% CI: 2.1-3.7; BCG group mean ΔOD: 2.4, 95% CI: 1.6-3.2; BCG-AdTBF group mean ΔOD: 2, 95% CI: 1.1-2.8).

## Discussion

Recently, our research group, in collaboration with other partners involved in human and animal TB vaccine development, proposed domestic goats as a new experimental TB model to be used in trials with the advantage of its simple husbandry and relatively low economical costs [27]. This model has been used herein to assess the efficacy of AdTBF, a new TB vaccine booster candidate containing four MTBC antigens, in combination with BCG (heterologous prime-boost procedure: BCG priming and AdTBF boosting). BCG-AdTBF induced improved protection compared to the protection observed in animals vaccinated only with BCG. Namely a reduction of the bacterial load and the severity of the pathology were observed.

A previous study using BCG and an Ag85A-monovalent-booster vaccine with the same vaccination strategy yielded similar results in terms of protection [24]. In both experiments, BCG-primed and adenoviral-boosted animals showed a mean volume of lung lesions of around 11 cm^3^ (less than 1% of lung involvement), and a bacterial load in pulmonary LN of approximately 3 Log_10_ (a reduction of 1 Log_10_ compared to unvaccinated animals). In contrast, despite experimental goats were of the same age in the two studies, BCG-AdTBF group showed a statistically significant increase in the weight gain compared to BCG and control groups, while there were no differences in weight gain among groups in the first study. Regrettably, due to logistical constraints BCG-AdTBF vaccination could not be directly compared in the present study with BCG-AdAg85A.

Unvaccinated goats, after challenge with *M. caprae,* progressed to clinical disease, while vaccinated goats (particularly BCG-AdTBF treated animals), showed mainly small, well-delimitated lesions, with microscopic features similar to granulomas found in human LTBI [31]. Goat and human lungs share essential morphologic characteristics, particularly in their intralobular septation by connective tissue (see Figure 5C). These structures may facilitate granuloma encapsulation and containment of mycobacterial spreading [32]. In contrast, small laboratory animal models often used in TB vaccine trials, such as mice, guinea pigs, rabbits and even macaques, do not show this lung compartmentalization [33].

Furthermore, goats have the advantage of reproducing the typical features of human active TB, such as lung cavitary lesions, which are frequently found in naturally infected goats [34] and are also induced upon experimental infection [27]. Even though cattle infected with *M. bovis* show similar lung granulomatous reaction and immune response to TB as humans do [35], evident cavitary lesions are difficult to induce experimentally [36].

To establish a relationship between the protective effect of vaccination and the lesion pattern, morphological features of small granulomas (SG, lesions with < 0.5 cm diameter) were investigated. Our aim was to determine if qualitative differences existed in a comparable lesion which could be found in the three groups of animals. The four stage classification of LN granulomas developed by Wangoo *et al*. for experimental *M. bovis* infection in calves [28] was followed for this purpose. Unvaccinated goats showed higher proportion of SG at initial developmental stages, while most SG from vaccinated animals showed characteristics of older, more evolved lesions, namely, high degree of necrosis, mineralization and partial or total encapsulation by connective tissue.

It could be speculated that poorly organized SG (found mainly in unvaccinated goats), without evident arrangement in layers of lymphocytes and macrophages, would easily progress to bigger granulomatous lesions. Indeed, all unvaccinated goats presented a variable number of LG, many of them liquefacted (shown at Figure 4).

At the microscopic level, we have introduced an additional parameter to evaluate lesion containment: the presence of small satellite granulomas (SatG) surrounding a central lesion. Histological examination suggests that SatG surrounding the main lesion will likely progress to new SG, ultimately evolving to coalescent larger lesions. The positive correlation between the extent of the pathology (volume of gross lesions and number of affected lung lobes) and the mean number of SatG (per animal) also supports this speculation.

SG in stages III and IV resemble lesions found in human LTBI [31]. Rapid granuloma encapsulation, preventing its liquefaction, cavitation and bacillary drainage to the airways, has been hypothesized as a key step for containing TB infection, and as an explanation for the induction of a human-like LTBI in a minipig TB model [32].

Additionally, the absence of big granulomatous lesions in BCG-AdTBF vaccinated animals is consistent with their lower systemic Th1 pro-inflammatory response post-challenge. Moreover, in some vaccinated individuals the gross lesion volume was proportionally more reduced than was the bacterial load found in its draining LN. This is consistent with the lower specific IFN-γ response against antigens secreted by the growing bacilli (i.e. ESAT-6, CFP-10 and Rv3615c) in vaccinated animals, suggesting that the bacterial load found might be mainly composed of an *in vivo* non-replicating bacilli subpopulation. This is in accordance to what occurs in TB murine model after short-term chemotherapy [37], used to study the nature of human LTBI. These observations suggest that vaccine-dependent pathogenesis of granuloma formation and development might occur in the goat TB model.

The cultured IFN-γ ELISPOT is an innovative method for measuring memory T-cell immunity, which has been successfully used as predictor of vaccine efficacy against infections caused by intracellular pathogens such as malaria [38] or TB [23]. Accordingly, we found a negative correlation between Ag85A-specifc SFC and the total volume of gross lesions (lungs and LN). These results endorse the measurement of specific IFN-γ-producing memory cells as a predictor of TB vaccine efficacy. Interestingly, only the cultured ELISPOT responses against Ag85A correlated significantly with protection (or negatively with extent of disease), with TB9.8 showing a non-significant trend, whilst responses to TB10.4 did not associate with disease at all. It is interesting to speculate that Ag85A, and to a lower degree TB9.8, are protective antigens, whilst TB10.4 may only have a minimal contribution to the overall protective immunity. However, this hypothesis needs to be tested using recombinant adenoviral vaccines expressing only single antigens. Unfortunately, it was not possible to measure Acr2 responses in this assay.

Concerning the immune correlates of infection assessed in the present study, the positive correlation of the specific IFN-γ levels to E/C and Rv3615c, as well as the specific antibody levels to the surface protein MPB83, with the severity of the pathology, are in agreement with other studies performed in calf and goats experimentally challenged with *M. bovis* and *M. caprae* respectively [24], [27], [39], [40].

Recently, the modified Vaccinia Ankara virus expressing antigen 85A (MVA85A), the first vaccine candidate after BCG, failed to confer statistically significant enhanced protection against TB disease or infection in 2794 South African infants, compared with BCG alone [41]. Despite this result, several other viral-delivered vaccines in clinical trials have shown improved protection in a number of animal models [21], [23], [42], [43]. Indeed, the results presented herein show enhanced protection of BCG-primed and AdTBF-boosted goats after the *M. caprae* challenge.

However, our findings are far away from the goal of obtaining a vaccine that prevents mycobacterial infection. In the last decade, the mucosal immunity in the respiratory airways has been receiving major attention in inducing protection against certain infectious diseases [44]. The availability of mucosal antibodies at the alveolar space might prevent bacilli reaching the lung from establishing the initial infection [45]. A future generation of vaccines could be focused on inducing pre-existing antibodies at the site of infection to eradicate the mycobacteria before they can hide within the target cells [46]. In this regard, BCG prime followed by mucosal administration of AdAg85A have already showed encouraging results in terms of protection against *M. tuberculosis* in small animal models [20], [21] and have shown similar immunogenicity than other routes of administration in cattle [22], [47]. Future trials using the experimental goat TB model could be directed towards: a) the use of *M. tuberculosis* challenge instead of *M. caprae* and b) the use mucosal administration routes of TB vaccine.

Following the “One health” approach, promoted by the WHO and OIE, the present study addresses the public and animal health challenge of TB control and eradication. Using goats as a large animal model of TB, we have shown an improvement of protection, when BCG vaccinated goats are boosted with AdTBF. These results support further evaluation of adenoviral-based multi-antigenic TB vaccines in clinical trials.
